# A guide to backward paper writing for the data sciences

**DOI:** 10.1016/j.patter.2021.100423

**Published:** 2022-01-03

**Authors:** Jon Zelner, Kelly Broen, Ella August

**Affiliations:** 1 Department of Epidemiology, University of Michigan School of Public Health, Ann Arbor, MI 48109, USA; 2 Center for Social Epidemiology and Population Health, University of Michigan School of Public Health, Ann Arbor, MI 48109, USA; 3 Pre-Publication Support Service (PREPSS), Ann Arbor, MI 48109, USA

## Abstract

In this perspective, we outline a set of best practices for the planning, writing, and revision of scientific papers and other forms of professional communication in the data sciences. We propose a backward approach that begins with clearly identifying the scientific and professional goals motivating the work, followed by a purposeful mapping from those goals to each section of a paper. This approach is motivated by the conviction that manuscript writing can be more effective, efficient, creative, and even enjoyable—particularly for early-career researchers—when the overarching goals of the paper and its individual components are clearly mapped out.

## The bigger picture

As the size and complexity of datasets and data science pipelines continue to grow, the challenge of crafting clear and engaging scientific writing to communicate data science research has also increased. This comes at a time when effective communication of complex models and results is increasingly critical: from climate science to epidemiological modeling to the social sciences and beyond, data science involving very large datasets and complex, computationally intensive modeling is increasingly at the heart of the global scientific, policy, and public conversation. This paper is aimed at helping data science researchers successfully craft original research papers describing their work.

## Teaser

In this perspective, the authors propose a backward approach to data science writing that begins with clearly identifying the scientific and professional goals motivating the work, followed by a purposeful mapping from those goals to each section of a paper. This approach is motivated by the conviction that manuscript writing can be more effective, efficient, creative, and even enjoyable—particularly for early-career researchers—when the overarching goals of the paper and its individual components are clearly mapped out.

## Introduction

Academic and applied research in data-intensive fields requires the development of a diverse skillset, of which clear writing and communication are among the most important.^1^ However, in our experience, the art of scientific communication is often ignored in the formal training of data scientists in diverse fields spanning the life, social, physical, mathematical, and medical sciences. Instead, clear communication is assumed to be learned via osmosis, through the practice of reading the work of others, writing up our own work, and receiving feedback from mentors and colleagues. What makes this way of learning frustrating for many researchers is that research papers in the quantitative sciences often have a relatively rigid format, and typically must be concise (i.e., 3,500 words or fewer), leaving little room for improvisation. In this perspective, we—a mid-career data science researcher in public health, advanced PhD student in quantitative epidemiology, and specialist in scientific writing—attempt to demystify the paper-writing process by providing a set of guiding questions that can be used to plan and revise each section of a paper and to help you position your paper to support your professional growth and goals.

Early-career data science researchers and practitioners may find themselves wondering how to work within the constraints of the research paper format to get their message across. In the worst case, the format is deadening, rather than freeing, and leads to papers that are as excruciating to read as they were to write. This problem becomes acute when the goal of publishing as communication is crowded out by the pressures of publishing for professional survival and advancement. When the volume and prestige of publications are prized over their value as tools of scientific communication—as they too often are^2^^,^^3^—the rigid format of the research paper can be just one more demotivating obstacle on a long and stressful career path. When the constraints and challenges of academic publishing are foregrounded, the process of writing can take on a sense of existential dread, with each unwritten manuscript section representing a new way to fail to achieve the standard needed to be successful.

### Writing for data science poses unique challenges

Data science is an integrative enterprise that brings the processes of data cleaning, manipulation, visualization, and other forms of processing under the same scientific tent as statistical analysis and mathematical modeling.^4^ Data scientists are often working at scale with “big data” for which the processes of procurement, storage, manipulation, and analysis is more complex than for smaller, less-complex datasets. This necessarily broadens the set of responsibilities the author of a data science paper has to explain a wide array of techniques, outputs, and results. To some extent, this mirrors age-old challenges in scientific writing: physical and natural scientists have long had to document their experimental setups, and social scientists present in-depth information on survey sampling and the collection of observational data. In fact, these fields have each become increasingly inflected by the challenges of working with large, complex datasets. So, while this guide is written with a data science audience in mind, we believe that the work of quantitative researchers in the sciences is increasingly that of disciplinary data science. We hope that the suggestions we provide will be applicable to the challenges of communicating increasingly complex analyses to as broad a scientific audience as possible.

What follows is a set of suggestions and questions to guide the process of writing up research for the data sciences. It is motivated by the strongly held conviction that the writing process should occur in parallel with the research process and that, in fact, the two are indistinct.^5^ We also provide as an appendix an annotated version of a recent manuscript from our own research using Bayesian hierarchical models with complex public health datasets.^6^ We use the annotation to show how the influence of these principles is reflected in a finished product. Our writing and professional advice is influenced by countless conversations and interactions with any number of colleagues, classmates, and mentors. While there are too many influences to thank, we are grateful for their cumulative wisdom, presented here in condensed form. We include citations to relevant resources and influences where possible (i.e., when the idea is presented in a paper, book, or blog post).

### Each section of a research paper has a well-defined purpose

The relatively rigid format of research papers is often a pain point in scientific writing: What goes in the introduction versus the discussion? How much detail should be in the methods section? When should I mention limitations of my analysis and why? How do I do this without it coming off as stiff and formulaic? After a while, the inflexibility of the form may reveal itself to be freeing, because it provides a structure you can use to ensure that your ideas are clearly organized and communicated in a way that ensures that as much of your intended audience as possible can read, build on, and replicate.^7^ The key thing to remember is that the sections of a research paper each have a distinct role to play in constructing and communicating your message, but they should also cohere and interact.^8^^,^^9^

Your paper is an extended argument about the relationship between a question (introduction), the way you decided to answer it (methods), what came out of it (results), what it all means (discussion), and what it implies for what is next (conclusion). If you start with a clear idea of what you want to accomplish with each of these sections, it will free you up to focus on your hypotheses, results, big ideas, and opinions.

### Work backward to make sure your intended message gets across

A well-crafted data science paper is a pedagogical tool that not only conveys information from author to reader but facilitates the understanding of complex concepts. This works in both directions: The paper-writing process is an opportunity for the writers to learn about and clarify their understanding of the topic in addition to communicating it to someone else. If we can accept the idea of this kind of writing as teaching, we can take a lesson from research and practice in the field of educational development, particularly the backward approach to curriculum design, introduced by Williams and McTighe in their book *Understanding by Design*^10^:

Our lessons, units, and courses should be logically inferred from the [learning outcomes] sought, not derived from the methods, books, and activities with which we are most comfortable. Curriculum should lay out the most effective ways of achieving specific results … the best designs derive backward from the learnings sought.

Under a backward-design approach, the overarching goals of a course are defined first, and then used to motivate and shape everything from the assignments students will complete, the nature and volume of reading material, and the way class meetings will be used to advance toward these goals. In this way of thinking, a course has a set of standard components—assignments, reading, class time—but the way in which they are devised and arranged is organized around supporting the learning goals of the class. The same approach can be applied to the construction of a research paper: even though most papers have the same sections (introduction, methods, results, discussion) early-career researchers may underestimate the amount of flexibility and room for creativity they have in using these components to achieve their scientific and professional development goals. The backward approach we lay out here is about starting at the end by answering the questions of “What do I want accomplish with this paper?” and scaffolding each piece to help serve those goals. This is contrasted with the more *ad hoc* forward approach most of us have learned to live with, in which we begin with the introduction and struggle through to the conclusion with the primary goal of simply finishing the manuscript.

## A guide to backward paper writing

While writing this guide, we struggled with what to call it: a checklist implies something proscriptive, a set of rigid “must-do” tasks, much as a pilot has a pre-flight checklist where each item is essential to a safe journey. However, the goal of writing for clear and impactful scientific communication is not served by ticking off externally imposed requirements without knowing why one is doing so.^11^ Good scientific writing should still tell a story that brings the reader along on a condensed version of your journey with a given project: why is the idea compelling and important? Why did you do what you did? What do you wish you could have done? What should you or someone else do next?^1^

Unfortunately, the hierarchical nature of many academic and non-academic research environments can result in scientific writers at all stages fixating on the voice of an internalized critic: the unsupportive professor, supervisor, or colleague who aggressively brushes you back if you get too confident. This can lead to defensive, apologetic writing through which we are pursuing the goal of not being criticized rather than the goals that brought us to the work in the first place. Our overarching objective in this piece is to help you keep focus on making an honest, affirmative, and enthusiastic argument in favor of the work you have done. To facilitate this, in what follows, we have constructed a set of questions to ask yourself and your coauthors throughout the process of planning, writing, submitting, and revising your paper. However, like any good guiding questions, they are only useful if you engage thoughtfully with them until they are answered to your satisfaction.

### Questions to help you plan your writing, reflect on your professional goals with the paper, and position your work

Before you get started, take some time to be sure you have a good sense of your answers to the following questions. Often, it can be helpful to make a fresh document in which you think out these sorts of high-level questions on the page. This sort of informal “pre-writing” is powerful because it can help you clarify your thoughts and professional goals^12^:

1. **Who is your intended audience?** This could also be described as, "What do you want to accomplish with your paper?" If you have a clear idea of who you hope will read your paper, it will help clarify the goals of your writing^13^: is the goal to showcase incremental improvement to existing approaches to a group of people who all specialize in the same area? Are you trying to reach a broad audience to convince them why some long-held idea is not quite right? Do you want to reach a specialist and non-specialist audience to contribute to policy or public conversation?
2. **What is the major idea/contribution of the paper?** If you have more than one, you either need to reconsider what your main idea is or think about splitting into two papers. The reason to do this is simple: you have not much space and not much attention from other people and you want to make the most of what you have. Sticking to one idea per paper^14^ is, in general, a good practice to ensure that your message comes through as clearly and impactfully as possible.
3. **What are your professional goals in writing this paper?** Your career stage and level of engagement with the topic can and should dictate the way you approach a paper. Is this the publication you want to showcase when looking for your next job? Is this part of building a research program on your own by testing out new ideas?
4. **What is the right outlet for this work?** Each journal has a core readership, as well as style, organization, and length requirements. Two important questions that should guide your choice of target journal are who the best audience is for your paper, and how much value you place on publishing in a prestigious journal (and your ability to tolerate the time investment and higher risk of rejection associated with aiming high) versus starting the process with an outlet that is less of a “reach” but can still let you reach the audience you want to connect with.^8^ Identifying the core readership of a journal, which is often laid out in bold print on the journal’s website, will help you reach those who are interested in your work and those who are poised to apply your findings.

Below, we describe each section of an academic journal article. We present these in the order they typically show up in a published paper, but we do not have hard-and-fast rules about the order in which they should be written: some writers prefer to start with the introduction section, and others prefer to start with the methods section. Experiment to find out what feels right for you.

## Introduction

5. **What is the problem your analysis is meant to address or solve?** Use the first paragraph or two to outline the scope and importance of the problem you are addressing. The importance may encompass the burden of disease (or other outcome), such as suffering and or mortality, and/or economic impact, as well as the scope or scale of the disease. The problem you articulate should be the specific knowledge gap that you will fill with your research, and the reader should come away with a good sense of why it is important to close it.
6. **What else has been tried?** This is the part where you get into the approaches that have been taken to this problem and the results that have come out of this.
  a. *No need to be negative*: This is not about why everything else is awful, but just what has already been accomplished, what others have taught us with their previous work. If you have prior work in this area, this is also a good place to cite it and highlight the continuity between your earlier research and what you are doing now.
7. **Nevertheless…** This is where you can identify the gap left by the previous work. For example, it may fit into one of these example categories:
  a. Previous analyses got at an *important question* but not with the kind of data you are using, which may be more detailed, contextually relevant, etc.
  b. Previous work used a *methodological approach* that was not able to get at some important dimension of the problem, over/under-stated variability in outcomes, etc.
  c. Earlier work did not address the *broader context* of the problem; i.e., too narrowly focused on a specific dataset or place and less on understanding the processes that cut across contexts.
8. **In this paper we will…** For the love of everything, please tell the reader what you are going to do before you do it. This is the point of departure for your reader on the journey that will be reading and metabolizing your paper: give them a map!
  a. *Use this part to mention the data you will use*; i.e. where it comes from and what outcomes measured by the data are the focus of your analysis.
  b. *Describe the analytic/statistical methods in brief*. Do not get into a ton of depth, just mention what you are going to do and provide a one-sentence justification. This allows the reader to have a sense of what is coming in terms of the analytic approach without getting into information that should be in the methods section. This kind of signposting is often very valuable for giving your reader a sense that they know where they are going when reading your paper rather than feeling lost or confused.

## Methods/data

The methods and data section can be tricky to write because it is where all the things you are trying to do converge, and where an interested reader or reviewer will spend a lot of their time trying to understand what you have done and verifying that the results and conclusions are justified. So, in this section we are a bit more detailed and proscriptive than in the others, since there is so much packed into a relatively small section of your paper:

1. **Introduce the methods and data in broad terms.** Use the first paragraph of the methods and data section to do some signposting; i.e., giving the reader a sense of the reasoning behind the study design, and motivation for using a particular dataset or focusing on a particular population to answer your particular question.
  a. *This applies even to methods papers:* In most cases, the introduction to the entire paper should be focused primarily on the applied problem at hand. If it is a more methods-focused or statistical paper, that may not entirely be the case. But even then, the opening of the methods section should focus on motivating the importance of the method for confronting real-world problems, rather than getting into the nuts and bolts of the approach.
2. **Go into detail on the data.** This section can and should be a bit more formulaic than the ones that came before. It really is as much “just the facts ma'am” as possible, but the trick is highlighting the facts about the data that are most relevant to what you are trying to do. The motivating question in putting this section together—which can include figures, tables, and written description—should be, "What does the reader need to know about the data to understand the results?”
  a. *What do the data measure?* Include outcomes, covariates, etc. If you have multiple sources (e.g. cases of a disease plus census population data), introduce both.
  b. *When/how were they collected? Who collected the data?* This is what is sounds like: give a sense of the provenance of the data. If they come from a larger/long-term study, refer to earlier analyses from the study. If a published study protocol exists, be sure to cite it. If the data are publicly available, link to them and be sure to include a timestamp showing when you retrieved them.
3. **What can go into a figure?** Visualizing data is almost always preferable to talking about them or presenting summary statistics in a table,^15^ but it requires care: think about what views of the data are most important for a descriptive figure that introduces the data and the problem (Figure 1). Remember that the point is *not* to make the reader as much of an expert about the data as you are, but to be familiar enough with the data to understand both the motivation for the analysis and the results when they come along. (See Nolan and Stoudt, 2021^1^ for a more in-depth exploration of the figure-making process.)

**Figure 1:**
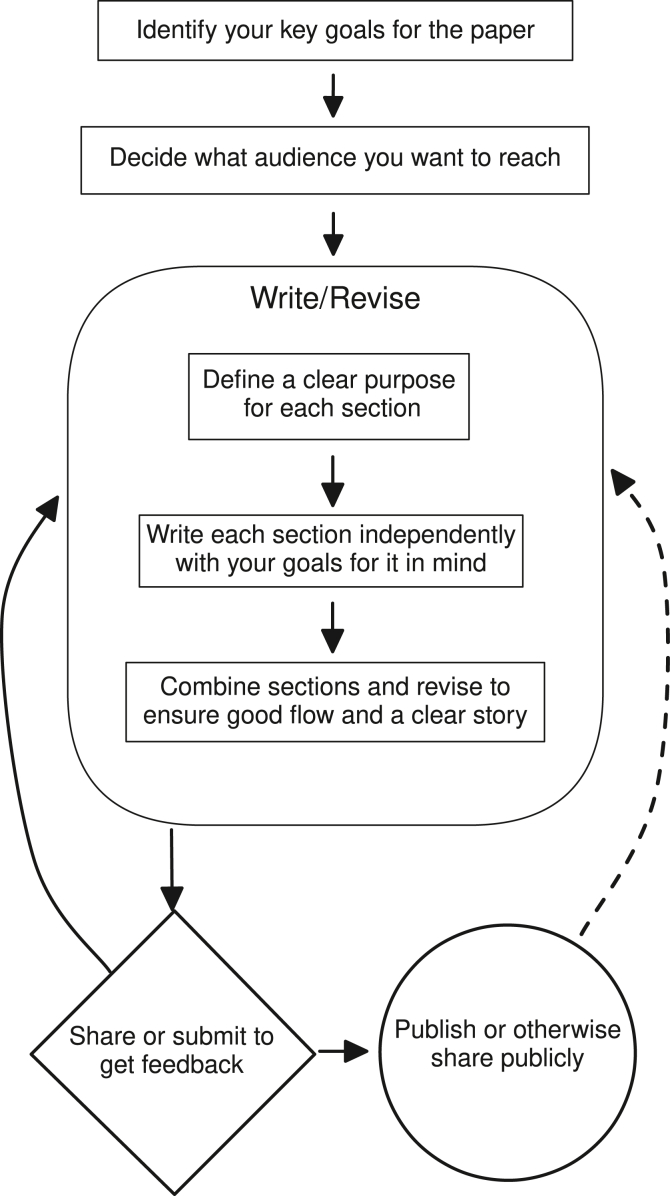
Schematic representation of the process of backward paper writing The high-level steps involved in the process of backward data science manuscript preparation. The square boxes at the top represent the important pre-writing steps in which you clarify the scientific and professional goals motivating your work. The rounded box represents the process of initial writing and revision. Once a draft is complete, the diamond box represents circulating the manuscript to colleagues and mentors for feedback, or submitting for publication, with the expectation that this will result in further revision and updating of your work. The circle represents the typical endpoint of the process: publication in a peer-reviewed outlet, sharing publicly via a preprint server, publishing online via an interactive notebook or app, or the many other ways in which data science research can be disseminated to relevant scientific communities and the public at large. Finally, the dashed arrow represents the potential for post-publication revision in response to feedback and critique or new data. While not required, this type of post-publication revision is increasingly common in data science fields, allows for greater transparency, and may increase the long-term relevance of the published work.
4. **Do I need a table?** Really, do you need a table?^16^ If you do, keep it short and sweet: present relevant information that is hard to get into a single figure (e.g., cramming the number of people in a study, the proportion in different age groups, and the distribution of individuals by sex, race/ethnicity, etc. into a figure may be needlessly complex and better accomplished in a table).

### Analytic methods

As methods-oriented people, we often want to get into a lot of detail here (we will speak for ourselves at least!). However, this is another moment where you need to consider your audience and goals carefully. You are probably doing a bunch of things in the paper that merit explanation in the methods section, but the ones you highlight in this section should fit the following criteria:

9. **What is the most important thing you are trying to do with your paper, and which methods are most crucial to understanding that?** This is another way of saying that you should focus on the model or set of models in the paper that are most important to understanding your results and advancing your overall agenda with the paper. You may have sub-analyses that provide extra detail but do not need to be outlined in depth in the main part of the manuscript. If it cannot all fit in the main text without it getting bloated, you can always refer the reader to a supplement containing this information.
10. **Which methods are important enough to describe is a function of your audience.** Think about what kind of paper this is and who you expect to read it; that will dictate what goes in this section. The more generic the audience is, the more likely it is that you will have a lot of the methods in a supplement, whereas, for a more specialist audience, you will probably go into more depth. For example, if your analysis uses a fancy new statistical technique and your goal is to highlight what more can be learned using this approach to other people working in the same area, then you might want to go into depth. However, if the point is to highlight the scientific importance of the result yielded by the method for a broader, non-specialist audience, you may save more of that detail for a supplement.
11. **How does what you are doing methodologically relate to the major questions of your analysis?** Remember that the methods section exists to give the reader the ability to understand and evaluate the results you are presenting to them. So, if the result of using a particular method is not going to be in the results in some way, it should not be in the methods section.

## Results

The point of the results section is to make the results of your analysis (descriptive statistics, assays, imaging results, parameter estimates, posterior predictions, model simulations, etc.) as clear as possible to the reader. The first part of that is figuring out the best way to communicate each relevant piece of information. We would boil this down into a set of simple suggestions:

12. **If it can be conveyed visually, do it!** Prefer figures over tables and in-text descriptions where you can.^15^^,^^17^ This is subject to limitations that force you to prioritize what goes into a figure and what does not: How many figures are allowed by the journal? Is it enough information to take up a whole figure or would briefly mentioning it in the text be a better use of space?
  a. *Figures and tables should stand on their own.* Reasonably informed readers should be able to get what is going on from looking at your figure and reading the legend, even if they have not read the rest of the paper. This is not a hard-and-fast rule, but if you work toward it you will ensure that the figures convey as much information as possible.
  b. *Each figure should make a clear point of its own.* If two separate figures convey overlapping information, try to eliminate one, or combine them both into a single panel (e.g., using left- and right-hand axes) or a multi-panel figure. Each full figure (i.e., with its own number) should touch on a single idea/result. For example, if you are reporting the results of an analysis that looks at multiple outcomes (e.g., the risk of developing a disease versus the risk of dying of it), it likely makes sense to put these in separate figures, much as you would place the discussion of them in separate paragraphs in the text.
  c. *Use your figures and results to tell a story.* Nolan and Stoudt suggest using a process of “storyboarding” in which you arrange your figures and tables in the order you think they make the most sense, and then write up your results in a way that takes you through each to tell the story of your analysis.^1^ Just like the process of storyboarding for TV and movies is an iterative one, the idea here is to give yourself maximum flexibility to re-arrange the pieces until you find a narrative that brings it all together.
13. **If you must make a results table, keep it small and simple.** Big, complex tables are where reader attention goes to die. If information is best conveyed by a table, be sure to include only the most essential information. When a table gets too big, it becomes easy to forget what its purpose is. By keeping it short and cutting out extraneous information, you are better able to keep the focus on your message.
14. **Use the beginning of the results section to hit the highlights in the figures and tables.** Imagine you are explaining the figures to someone: what is the most important thing you want them to get from the figure? Talk about that in the beginning of the results section. Whatever you do, do not recapitulate entire figures and tables. They are part of your results, you can and should refer to them, but they should be complementary to what you are writing here, not duplicative or completely disjointed from that.
15. **Use the remaining text of the results section to provide information*****not*****in the figures or tables.** What else is important to know that is not captured by a figure or table? Is there a single estimate from a side analysis that fills in the story but does not warrant a figure or table on its own?

### Ethical reporting of methods and results

Often, when we investigate a question, we attempt multiple analytic approaches before deciding on the one presented in the final paper. This is in the nature of the research process and is not inherently problematic. However, it can present a conundrum when the time comes to write up your paper: the short format and the need to tell a compelling story can result in papers that make a complex data science project appear to have occurred in one straight line from hypothesis to data to methods to results. It is important to think carefully about which of these detours is important enough to include in the main text versus the supplementary materials or some other product associated with your paper.^18^

You can also use the data science ecosystem to your benefit. Rather than fighting the constraints of the research paper structure, it can help to think of your paper as a single node in a larger network of outputs that provide transparency and reproducibility. For example, by releasing the code to complete your analysis, and the data underlying it if it is possible to do so without sacrificing privacy,^19^ you give other researchers the ability to assess your research products directly by re-running the code used to generate your results. However, you could also describe your process in greater depth in a blog post or a standalone essay submitted to a preprint server like Arxiv.org. You could release a step-by-step walk-through of your analysis using Jupyter notebooks, Shiny apps for R, or any other tool that facilitates more interactivity than a static PDF.

This kind of transparency allows you to illustrate and defend your approach and to also explain how you accounted for prior explorations in your presentation of results. This can help you increase confidence that your major results are not a reflection of data-driven hypothesis-testing, i.e., p-hacking or a trip through the “garden of forking paths,” in which researchers make many comparisons before deciding on the ones to present in a manuscript.^20^

## Discussion

The discussion is where you get to be a bit more expansive and opinionated. As ever, though, think about how each of the things you put in here will affect what you are trying to accomplish. We tend to think of the discussion as having its own subsections^7^^,^^9^ that roughly look like this:

16. **First paragraph of discussion: Summarize, summarize, summarize.** What did you accomplish? How did your results relate to the problem/hypotheses you laid out in the introduction? If I had not read the introduction, methods, or results, and just opened it to the discussion and read this paragraph, I should be able to get what you did.
17. **Second paragraph of discussion: Sell the product.** This is it: time to make the affirmative case for what you did. Why is it important? Why was your approach well suited to answering the question? What gaps have you worked toward closing that you highlighted in the introduction? Again, there is no need to be negative about other work, just show how you have moved the research forward in some meaningful way.
18. **Third paragraph: Limitations.** Writing this part can be uncomfortable or scary sometimes because it seems like you are being asked to undermine your work. However, a well-written limitations paragraph adds to your credibility by showing you have thought about what can and cannot be learned using the data available and the methods employed. Try to answer the question of, "What question might someone else want to answer that my paper does not/cannot address?" Limitations are reasonable stopping points for what you are trying to do that demarcate the boundaries of your analysis. Sometimes a question that should have been asked was not included in a survey, or an instrument malfunctioned during an experiment that was too costly or time consuming to be repeated. Being forthright about these limitations is also a helpful reminder to your readers (and yourself) that you cannot solve every problem, and that your results reflect the limits of the tools and data available to you.
  a. *Future directions.* Once you have given the reader a sense of what your analysis does and does not do, you can talk about how you, or someone else, can take the next step to transcend the limitations you have highlighted. This is an opportunity to plant your flag on the things you want to do next or that you have just started doing. You can use this as a form of motivation and accountability: What did I not do here that I want to do next?
19. **Fourth paragraph:Conclusions** (sometimes part of the Discussion, sometimes a separate section of the paper). This is where you tie everything together: What does it all mean and why does it matter? What comes next? What is the big point you want to stick with the reader? Here is where you can get a bit more opinionated, editorialize a bit, and even make some tentative extrapolations or predictions about the future.

### Accept—and anticipate—the process of revising your work

The process of submission, rejection, and resubmission is often deeply discouraging to early-career researchers. A common misconception is that the best papers sail through review without revision or critique from reviewers, editors, and colleagues. However, it is in fact this process of feedback and revision that often makes good papers great.^21^ It cannot be reiterated enough that critiques or suggestions for improvement to your work do not imply that you have failed and that it is unpublishable.^22^^,^^23^ We hope that following a process like the one outlined here, whether you are an early-, mid- or late-career researcher, will allow you to remain motivated throughout what can be a stressful and oftentimes perplexing process.

### Make it your own

We hope that the outline provided here is helpful and provides a roadmap, or at least a set of guardrails, for your writing. It is important to remember that even scientific writing is a personal and creative process. Our initial goal in writing this guide was to make that process easier, less painful, and even a bit fun for ourselves. To facilitate the adoption and customization of the approach outlined here by data science researchers across the scientific spectrum, several versions of these questions are available at https://github.com/epibayes/paper-template. In addition to providing a version in Word, versions in Markdown and RMarkdown are available as well. This way, you can download a copy and begin filling in the sections as you go, using the questions as a scaffold for your research and writing. You are also invited to edit and remix this guide based on your own experiences and interests, with the hope that you will share your insights with colleagues and the next generation of researchers.

## Supplemental information

Document S1. Data S1

Document S2. Article plus supplemental information
